# Legionella Coinfection in a Patient With COVID-19 Pneumonia

**DOI:** 10.7759/cureus.17356

**Published:** 2021-08-21

**Authors:** Yogesh Subedi, Christopher J Haas

**Affiliations:** 1 Medicine, MedStar Union Memorial Hospital, Baltimore, USA; 2 Medicine, MedStar Franklin Square Medical Center, Baltimore, USA; 3 Medicine, MedStar Harbor Hospital, Baltimore, USA; 4 Medicine, George Washington University School of Medicine and Health Sciences, Washington, DC, USA

**Keywords:** covid-19, coinfection, legionella, dexamethasone, remdesivir, azithromycin, rheumatoid arthritis

## Abstract

Bacterial superinfection is a well-reported complication of viral pneumonia leading to significantly increased morbidity and mortality. Such superinfections have been reported in patients with pathogenic coronaviruses including severe acute respiratory syndrome (SARS) and the Middle East respiratory syndrome (MERS), but there are scant reports pertaining to superinfection in the context of coronavirus disease 2019 (COVID-19). We report a case of a middle-aged man who presented with worsening shortness of breath in the context of COVID-19 complicated by superimposed *Legionella pneumophilia* pneumonia. This case serves to highlight the possibility of bacterial superinfections and to be aware of such possibilities when patients are not responding to standard courses of treatment for COVID-19 as quick clinical deterioration is likely to develop.

## Introduction

At the end of 2019, a novel coronavirus was identified as the cause of a cluster of pneumonia cases in Wuhan, China [1]. Coronavirus disease 2019 (COVID-19) has not only caused physical, social, or psychological distress but also caused cardiac complications in non-COVID-19 patients due to the current pandemic [2]. Up to 30% of patients infected with severe acute respiratory syndrome (SARS) were diagnosed with secondary bacterial coinfection with associated increased disease severity [3]. Reports of co-infection with respiratory pathogens in the current coronavirus pandemic (COVID-19) is increasing throughout the world [4]. Lansbury et al. reported that 7% of hospitalized COVID-19 patients had bacterial pneumonia and the incidence was highest amongst ICU patients [5]. Zhou et al. showed that 27 (50%) of 54 non-survivors had secondary infections in a study of 191 patients in China [6]. Common co-pathogens in COVID patients included bacteria: *Streptococcus pneumoniae*, *Staphylococcus aureus*, *Klebsiella pneumoniae*, *Mycoplasma pneumoniae*, *Chlamydia pneumonia*, *Legionella pneumophila*, and *Acinetobacter baumannii*; Candida species and *Aspergillus flavus* among fungus; and viruses including influenza, parainfluenza, coronavirus, rhinovirus/enterovirus, metapneumovirus, influenza B virus, and human immunodeficiency virus [4].

## Case presentation

A 58-year-old gentleman with a history of hypertension, hyperlipidemia, obesity, rheumatoid arthritis (RA), tobacco abuse, and opiate abuse on methadone presented to the emergency department (ED) with a worsening nonproductive cough and shortness of breath of four days duration. His symptoms were associated with generalized myalgia, fatigue, diaphoresis, and non-bloody diarrhea. He denied sick contacts but was employed as a maintenance manager with a focus on air conditioner repair.

To note, he was originally diagnosed with osteoarthritis in the context of bilateral knee pain, associated swelling, and morning stiffness, however, was subsequently diagnosed with RA that was managed with chronic daily prednisone (30 mg) for three years before transitioning to adalimumab, a tumor necrosis factor-alpha (TNF-a) inhibitor. He received his first infusion one week prior to the hospital presentation.

**Figure 1:**
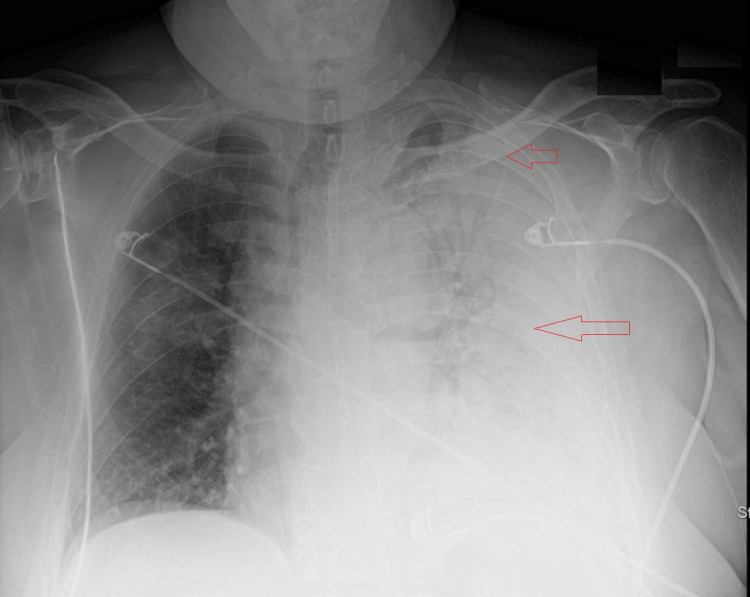
Chest X-ray demonstrates opacification of the entire left hemithorax with associated air bronchograms.

**Figure 2:**
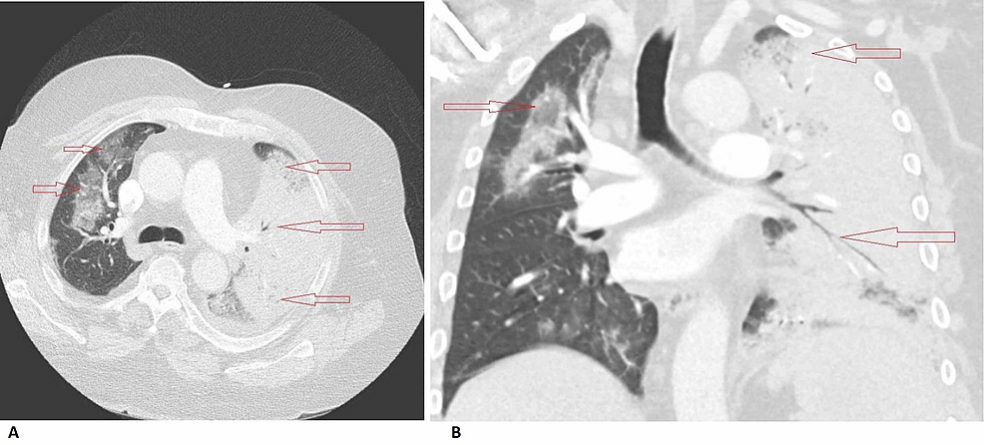
Chest CT demonstrates complete opacification of the left lung fields with diffuse pneumonia, trace pleural effusion, and multiple ground glass alveolar infiltrates involving the right upper, middle, and lower lobes in (A) transverse view and (B) coronal view.

In the ED the patient was afebrile, normotensive (132/82 mmHg), but markedly tachycardic (157 beats per minute), tachypneic (30 breaths per minute), and hypoxic to 72% on room air. There was a minimal improvement with nasal cannula and nonrebreather, and the patient was initiated on heated high flow nasal cannula (55 L; 100% FiO2) with improved saturation to 95%. Physical examination revealed an obese gentleman with cushingoid features (buffalo hump, abdominal striae) and bilateral lower limb pitting edema. Cardiac examination was unremarkable and pulmonary auscultation revealed bilateral decreased breath sounds in the lower lobes. Joints evaluation revealed mild bilateral knee swelling and effusions without additional joint deformities. Laboratory diagnostics demonstrated mild leukocytosis with a slight neutrophilic predominance, but no evidence of anemia or thrombocytopenia. The metabolic panel was notable for an elevated blood urea nitrogen, creatinine (baseline < 1), and transaminitis with elevated inflammatory markers (Table 1). Urinalysis was unremarkable with the exception of microscopic hematuria. Nasopharyngeal swab for COVID-19 RNA polymerase chain reaction (PCR) and urinary legionella antigen were positive. Diagnostic imaging included a chest radiograph that demonstrated extensive consolidation throughout the left lung field with air bronchograms involving the upper lobe, lower lobe, lingula, and right upper lobe (Figure 1). Chest CT was negative for pulmonary embolism but revealed complete opacification of the left lung fields with diffuse pneumonia and associated air bronchograms, trace pleural effusion, and multiple ground glass alveolar infiltrates involving the right upper, middle, and lower lobes (Figure 2).

**Table 1:** Laboratory analysis. CRP, C-reactive protein; LDH, lactate dehydrogenase; WBC, white blood cell; BUN, blood urea nitrogen; AST, aspartate aminotransferase; ALT, alanine aminotransferase

Laboratory analysis	Level	Normal range
CRP	444 mg/dL	0-3 mg/dL
D-Dimer	6.43 mcg/mL	<0.58 mcg/mL
Ferritin	1350 ng/mL	28-365 ng/mL
LDH	800 u/L	87-241 u/L
WBC	13.8 k/uL	4-10.8 k/uL
Absolute lymphocyte count	0.5 k/uL	0.6-4.9 k/uL
Absolute neutrophil count	12.7 k/uL	1.7-8.1 k/uL
Albumin level	2.3 g/dL	3.5-5 g/dL
BUN	40 mg/dL	9-20 mg/dL
Creatinine	1.81 mg/dL	0.5-1 mg/dL
AST	131 u/L	<40 u/L
ALT	55 u/L	<40 u/L
Lactic acid	3.5 mmol/L	0.5-2 mmol/L

 

The patient was admitted to the intensive care unit (ICU) for acute hypoxic respiratory failure in the setting of COVID-19 and Legionella pneumonia and initiated on dexamethasone (10 mg, daily; 10 days), remdesivir (daily; 5 days), azithromycin (500 mg, daily; 14 days), and ceftriaxone (1 g, daily, 7 days). It took 18 days to wean him to room air and prior to discharge, repeat COVID testing was negative. He was discharged on prednisone 30 mg daily for his RA and sulfamethoxazole-trimethoprim (Bactrim double strength, DS 800-160 mg) thrice weekly for pneumocystis carinii pneumonia (PCP) prophylaxis.

## Discussion

COVID-19 is a heterogeneous disease typified by a myriad of clinical presentations that range from asymptomatic to severe illness characterized by hypoxemic respiratory failure or multiorgan failure. While COVID-19 generally presents as a primary respiratory infection, with symptoms of cough, fever, myalgias, and shortness of breath, as is typical of many other respiratory viruses, many organ systems may be affected, resulting in a diverse array of symptoms [7-8]. Legionella pneumonia may have a similar presentation, with patients typically experiencing fever, nonproductive cough, dyspnea, headache, myalgia, and diarrhea [9]. Laboratory diagnostics often demonstrated hyponatremia, hypophosphatemia, transaminitis, microscopic hematuria, and elevated inflammatory markers [erythrocyte sedimentation rate (ESR), C-reactive protein (CRP), ferritin], [10] all similarly seen in COVID-19, potentially confounding a secondary/ superimposed infection.

In this report, we present a patient with typical symptoms (worsening shortness of breath, cough, myalgia, fatigue, and diarrhea), laboratory markers [CRP, lactate dehydrogenase (LDH), ferritin, transaminitis], and imaging (ground glass opacification) consistent with a diagnosis of COVID-19 [11]. However, the patient demonstrated complete left lung consolidation with associated air bronchograms and parapneumonic effusion, a finding considered to be atypical for COVID-19. His job as an air condition repair personal and positive urine Legionella antigen suggested a superimposed infection, which prompted dual management for COVID-19 and Legionella pneumonia. He was treated with a standard regimen for COVID-19 and a prolonged antibiotic course for his superimposed infection given his underlying immunocompromised status.

During the 2009 influenza pandemic, one in four severe cases of influenza A (H1N1) were associated with superimposed infection, leading to a significant increase in morbidity and mortality, with Streptococcus pneumoniae the most commonly identified superimposed bacteria [12]. Opportunistic infections due to bacterial, mycobacterial, invasive fungal, viral, parasitic, or other opportunistic pathogens including aspergillosis, blastomycosis, candidiasis, coccidioidomycosis, histoplasmosis, legionellosis, listeriosis, pneumocystosis, and tuberculosis have been reported with TNF blockers, including adalimumab [13-14]. The patient presented in this case had a history of RA managed with chronic prednisone and was recently transitioned to the tumor necrosis factor-alpha (TNF-a) inhibitor, adalimumab one week prior to hospital presentation.

Hypercytokinemia is a hallmark of COVID-19 [15] and is hypothesized to contribute to pulmonary pathology by enhancing capillary leakage, recruitment of inflammatory lymphocytes, neutrophils, and macrophages [16]. Blockade of the TNF pathway decreases IL-1 and IL-6 concentrations in patients with active RA [17] and also downregulates adhesion molecules and vascular endothelial growth factor [18], suggesting a potential modulating role in COVID-19 mediated pulmonary pathology. The pattern of pro-inflammatory effector cytokines in the alveolar membranes during severe COVID-19 shares similarities with the primary cytokines targeted in the treatment of RA, with both diseases leading to profound inflammation and tissue destruction. Thus while biological therapies like adalimumab and tocilizumab may have some beneficial effect on COVID-19 mediated lung inflammation, the complexity of the disease, the resultant cytokine storm, and temporal elaboration of a complex interplay of cytokines and chemokines, warrants further research not only into the mechanisms of COVID-19 pathology but also potential therapeutic targets [15, 19].

## Conclusions

Superimposed bacterial infections remain a distinct and overwhelmingly concerning sequelae of COVID-19 pneumonia that may contribute to significant morbidity and mortality in an already challenging disease. In cases of underlying immunosuppression with chronic steroids or disease-modifying therapies on the background of a previously primed immune system due to autoimmune, hyperinflammatory conditions, such as RA, the clinical course may be further complicated. Clinicians should remain aware of the potential for co-infection in the context of COVID-19 pneumonia. The decision to continue disease modifying anti rheumatoid drugs or immunosuppressive agents including biologics in the setting of COVID-19 infection with RA needs further research.
